# Whole-genome sequencing and characterization of an antibiotic resistant *Neisseria meningitidis* B isolate from a military unit in Vietnam

**DOI:** 10.1186/s12941-019-0315-z

**Published:** 2019-05-06

**Authors:** Thach Xuan Tran, Trang Thu Le, Long Phi Trieu, Christopher M. Austin, Dong Van Quyen, Huong Minh Nguyen

**Affiliations:** 1 Laboratory of Molecular Microbiology, Institute of Biotechnology, Vietnam Academy of Science and Technology, 18 Hoang Quoc Viet Street, Cau Giay District, Hanoi, Vietnam; 2 Laboratory of Microbiology, Military Institute of Preventive Medicine, 21 Trung Liet Street, Dong Da District, Hanoi, Vietnam; 3 Centre for Integrative Ecology, School of Life and Environmental Sciences, Deakin University, 75 Pigdons Rd, Waurn Ponds, Geelong, VIC 3216 Australia; 4 Pharmacological, Medical and Agronomical Biotechnology (PMAB) Department, University of Science and Technology of Hanoi, 18 Hoang Quoc Viet Street, Cau Giay District, Hanoi, Vietnam

**Keywords:** *Neisseria meningitidis*, Next generation sequencing, Epidemiological characterization, Antibiotic resistance, Antigen sequence typing, Vietnam

## Abstract

**Background:**

Invasive meningococcal disease (IMD) persists in military units in Vietnam despite the availability of antibiotics and vaccines. A hindrance to reducing the incidence of IMD in Vietnam is a lack of molecular data from isolates of the causative agent, *Neisseria meningitidis* from this country. Here, we characterized key genetic and epidemiological features of an invasive *N. meningitidis* isolate from a military unit in Vietnam using whole-genome sequencing.

**Methods:**

*Neisseria meningitidis* was isolated from a conscript admitted for meningitis and tested against seven antibiotics. DNA from the isolate was extracted and sequenced using the Illumina HiSeq platform. Denovo assembly and scaffolding were performed to construct a draft genome assembly, from which genes were predicted and functionally annotated. Genome analysis included epidemiological characterization, genomic composition and identification of antibiotic resistance genes.

**Results:**

Susceptibility testing of the isolate showed high levels of resistance to chloramphenicol and diminished susceptibility to ampicillin and rifampicin. A draft genome of ~ 2.1 Mb was assembled, containing 2451 protein coding sequences, 49 tRNAs and 3 rRNAs. Fifteen coding sequences sharing ≥ 84% identity with known antibiotic resistance genes were identified. Genome analysis revealed abundant repetitive DNAs and two prophages. Epidemiological typing revealed newly described sequence type, antigenic finetype and Bexsero^®^ Antigen Sequence Typing (BAST). The BAST profile showed no coverage by either Bexsero^®^ or Trumenba^®^.

**Conclusions:**

Our results present the first genome assembly of an invasive *N. meningitidis* isolate from a military unit in Vietnam. This study illustrates the usefulness of whole genome sequencing (WGS) analysis for epidemiological and antibiotic resistance studies and surveillance of IMD in Vietnam.

## Background

Invasive meningococcal disease (IMD) was first reported in the early 1800s as an emerging infectious disease [1]. IMD starts without clear symptoms but progresses rapidly into meningitis with, at times, septic shock that can be fatal [2]. Caused by the meningococcus bacterium, *Neisseria meningitidis*, IMD can generally be effectively prevented by vaccination and, given timely diagnosis, treated by appropriate antibiotics [3]. However, due to the lack of molecular characterization and proper epidemiological surveillance, fatality rates and disease sequelae can be high and severe [4, 5]. *Neisseria meningitidis* asymptomatically colonizes the nasopharyngeal mucosa of about 10% of the human population [6], but this rate drastically increases in certain living environments such as college dormitories or military units [7, 8]. IMD is relatively frequent in military units Vietnam, where prophylaxis and case treatment is based mainly on the experience of on-site medical personnel, mostly without the aid of molecular characterization and diagnosis. The drug of choice for treatment of IMD used to be penicillin and chloramphenicol [9], however due to resistance to these and some other antibiotics [10], third-generation cephalosporins, such as ceftriaxone and cefotaxime, are now the most common choice of treatment. These drugs, however, are often not readily available in many medical units in Vietnam.

Immunologically, *N. meningitidis* is divided into 12 serogroups, with most invasive strains belonging to serogroup A, B, C, W, X and Y. Strains can be further divided into serotypes and serosubtypes based on their outer-membrane antigens PorB and PorA, respectively [2]. A multi-locus sequence typing (MLST) scheme was first suggested by Maiden and colleagues in 1998 [11] utilizing seven house-keeping genes *abcZ, adk, aroE, fumC, gdh, pdhC* and *pgm* is now widely used to classify *N. meningitidis* isolates into sequence types (STs). Closely related STs can then be clustered together into groups and clonal complexes (CCs), with hypervirulent isolates often falling into several distinct CCs [12]. PubMLST is currently the biggest public database that catalogs genetic data and isolate provenance of the *Neisseria* genus [13].

Sanger sequencing provided the means for obtaining the initial genome sequence for a strain of *Neisseria meningitidis* [14]. However, it wasn’t until next-generation sequencing technologies were developed and evolved to allow far more rapid and inexpensive genomic studies, including those focused on *N. meningitidis*, that a fundamental understanding of the nature of metabolism, gene expression, and genetic variability within and between species could be obtained [15]. Recently, the number of available *N. meningitidis* genome sequences was reported to be 13 985 and still growing steadily [16]. Genomic studies of *N. meningitidis* have revealed important mechanisms underlying metabolic pathways [17], outbreak detection [18] and disease surveillance [19].

In this study, we used whole-genome sequencing (WGS) to assemble the genome of an invasive *N. meningitidis* isolate from a conscript in a military unit in Vietnam. Sequences obtained from WGS were annotated to determine the genetic and epidemiological characteristics of this Vietnamese isolate, together with an investigation of antibiotic resistance and vaccine coverage. This type of molecular characterization is needed for accurate IMD monitoring and surveillance and effective vaccination and for developing recommendations not only for military units but also other environments and communities in Vietnam.

## Materials and methods

### Bacterial isolation and typing

An invasive *N. meningitidis* strain was isolated at the Laboratory of Microbiology, Military Institute of Preventive Medicine, Hanoi from cerebrospinal fluid (CSF) of a conscript presenting to Military Hospital 108 with sepsis and meningitis symptoms in 2014. In total, 2 ml of cerebrospinal fluid was collected from the patient before administration of ceftriaxone, maintained at 35 °C and transferred to the laboratory within one hour. Sample was centrifuged at 5000 rpm for 10 min and sediment was spread on Mueller Hinton (MH) chocolate agar (Difco, USA). Gray colonies were observed after 24 h of incubation at 37 °C supplemented with 5% CO_2_. Two to three colonies were selected for Gram staining along with strain identification using Vitek^®^ 2 Compact system (bioMerieux, France) as per manufacturer’s instructions. Identified isolates were maintained on MH chocolate agar for immediate testing or stored at − 70 °C.

Serogroup typing and multi-locus sequence typing (MLST) was done according to previously described standard methods from the CDC laboratory manual for the diagnosis of meningitis [20].

### Antibiotic susceptibility assay

To determine antibiotic susceptibility, isolate stored at − 70 °C were transferred to MH chocolate agar and recovered at 37 °C and 5% CO_2_ for 24 h. A total of seven antibiotics were tested, consisting of ampicillin, ciprofloxacin, cefotaxime, ceftriaxone, rifampicin, meropenem and chloramphenicol. MIC values were determined using E-test strip (bioMerieux, France) following manufacturer’s guideline and susceptibility was interpreted according to CLSI 2018 breakpoints [21].

### Genomic DNA extraction and sequencing

Genomic DNA from the *N. meningitidis* DuyDNT strain was extracted using GeneJET Genomic DNA Purification Kit (Thermofisher Scientific) in accordance with the manufacturer’s instruction. The quality of DNA was assessed using an Agilent Technologies 2100 Bioanalyzer and sequenced using the Illumina HiSeq 4000 system (Macrogen). Raw images for system control were generated by HCS (HiSeq Control Software v3.3) and bases were called by RTA software (Real Time Analysis. v2.7.3). The BCL (base calls) binary was converted into FASTQ utilizing Illumina package bcl2fastq (v2.17.1.14).

### Genome assembly and annotation

Raw reads was preprocessed to remove adapters and low quality reads using Trimmomatic (parameters: ILLUMINACLIP:2:30:10 LEADING:3 TRAILING:3 SLIDINGWINDOW:10:30 MINLEN:100) [22]. FASTQC was then used to determine sequence quality before and after preprocessing [23]. Reads passed filtering were used for a de novo assembly using Velvet and VelvetOptimiser [24, 25], with contigs shorter than 500 bp being discarded. To assess the completeness of the genome, Benchmarking Universal Single-Copy Orthologs v.3.0.2 (BUSCO) was used [26] and scaffolds were roughly ordered and oriented using MeDuSa with genomic sequence of *N. meningitidis* MC58 strain served as the reference genome [27]. The resulting scaffolds were submitted to PATRIC web server [28] for protein prediction and annotation.

### Genome analysis

Genomic DNA sequence were submitted to PubMLST for identification of sequence type, antigenic finetype and Bexsero Antigen Sequence Typing following the database’s criteria. Complete genome sequences of *N. meningitidis* MC58, *N. lactamica* Y92–1009 and *N. gonorrhoeae* NCCP11945 were retrieved from the NCBI public database. Tandem Repeats Finder [29] was used to identify total number of repetitive DNA motifs in the genome. Frequency of specific repetitive motifs were analyzed by fuzznuc package of EMBOSS server.

Annotated amino acid sequences from DuyDNT genome were submitted to ResFinder 3.0 (https://cge.cbs.dtu.dk/services/ResFinder/), CARD (https://card.mcmaster.ca/analyze/rgi), and ARDB (https://ardb.cbcb.umd.edu/) to detect coding sequences involved in antibiotic resistance. PSI-BLAST were then used to find homologues to each identified coding sequence.

## Results and discussion

### Case description and isolate characterization

A 21-year old male conscript presented to his unit’s medical center with headache, tiredness and fever at midnight in June, 2014. One hundred and fifty minutes later, he developed meningitis symptoms including nausea, drowsiness, confusion, stiff neck and Kernig sign. He was given 1000 mg of Amoxicillin, 1000 mg of Paracetamol and an IV dose of Ringer’s lactate solution. By 9:30 A.M., he was transferred to the emergency department of Military Hospital 108 showing symptoms of blood sepsis. He was diagnosed with meningitis and sepsis, and was treated with ceftriaxone at the dose of 1 g, four times a day. His cerebrospinal fluid was collected and a *N. meningitidis* culture was grown in 12 h. The obtained *N. meningitidis* isolate, designated DuyDNT, was typed and identified to belong to serogroup B. The patient recovered successfully after treatment.

DuyDNT isolate was revealed to be a novel sequence type and assigned an ST 13074 by PubMLST, a public database that catalogs genetic data and isolate provenance of the *Neisseria* genus [13]. By sequencing the seven housekeeping genes *abcZ, adk, aroE, fumC, gdh, pdhC* and *pgm* utilized in the MLST scheme, every *N. meningitidis* isolate can be assigned a sequence type (ST) and placed into groups and clonal complexes based on evolutionary relatedness [11]. Besides DuyDNT, only two other isolates of ST 13074 have been found, both were carrier strains identified in Vietnam in 2017 (submitted to PubMLST by our group from unpublished data). ST 13074 shared ≥ 5 identical alleles at seven typed loci with three other STs (1576, 11013 and 13455), thus making it the central ST of this group by PubMLST group’s definition (Table 1). Except for isolate M3369 (ST 1576) that was identified in Italy from an invasive case, all other isolates of this group have only been detected in Vietnam, during the period from 1986 to 2017. This group do not form any clonal complex with any known isolates to date.

**Table 1:** Epidemiological characterization of DuyDNT isolate and other related isolates worldwide

Isolate	Country	Year	Status	Capsule group	ST	PorA VR1	PorA VR2	FetA VR	fHBP	NHBA
M3369	Italy		IMD	NA	1576					
Mrs2008309	Vietnam	2008		B	1576	22	9			
Mrs2008310	Vietnam	2008		B	1576	22	26			
Mrs2008311	Vietnam	2008		NG	1576	19	15–39			
Mrs2008312	Vietnam	2008		NG	1576	19	15–39			
13,515	Vietnam	1986	IMD	B	11,013					
DuyDNT	Vietnam	2014	IMD	B	13,074	22–25	14–32	F4–6	31	16
17,088	Vietnam	2017	C	B	13,074	22–25				
17,090	Vietnam	2017	C	B	13,074	22–25	14			
Bach	Vietnam	2013	IMD	B	13,455	19	15			

*NA* information not available, *NG* non-groupable, *IMD* invasive meningococcal disease, *C* carrier

### Antibiotic susceptibility testing

In Vietnam, antibiotics are still the first choice for treatment and prophylaxis against bacterial meningitis. However, Vietnam has become a hotspot for antibiotic resistance due to excessive and unregulated use of antibiotics [30]. Diminished susceptibility and resistance to antibiotics was observed in *N. meningitidis* before [10], posing a threat to the success of treatment since meningitis is an acute infection. To examine the extent of antibiotic resistance of DuyDNT isolate, we performed antibiotic susceptibility testing according to The Clinical and Laboratory Standards Institute (CLSI) 2018 guideline. The antibiotics tested were listed in Table 2, consisted of ones commonly used in meningitis prophylaxis (ampicillin, ciprofloxacin and rifampicin) and therapy (chloramphenicol and meropenem) in Vietnam, as well as ones more frequently used in therapy in developed countries (cefotaxime and ceftriaxone).

**Table 2:** Antibiotic susceptibility result of *N. meningitidis* isolate from Vietnam

Antibiotics	MIC breakpoints (μg/ ml)^a^			MIC value (μg/ ml)	Susceptibility
	S	I	R		
Ampicillin^b^	≤ 0.12	0.25–1	≥ 2	0.5	I
Ciprofloxacin^b^	≤ 0.03	0.06	≥ 0.12	0.008	S
Cefotaxime	≤ 0.12	–	–	0.016	S
Ceftriaxone	≤ 0.12	–	–	0.004	S
Rifampicin^b^	≤ 0.5	1	≥ 2	1.5	I
Meropenem	≤ 0.25	–	–	0.094	S
Chloramphenicol	≤ 2	4	≥ 8	256	R

^a^According to CLSI 2018 guideline

^b^Antibiotics currently used in meningitis prophylaxis in Vietnam

Resulting MICs showed that the DuyDNT isolate was still susceptible to most broad-range antibiotics, such as ciprofloxacin, cefotaxime, ceftriaxone and meropenem. In contrast, diminished susceptibility was observed toward ampicillin and rifampicin. This might be due to the fact that ampicillin and rifampicin are available in almost all military units and widely used for treatment and prevention of all nasal and upper respiratory tract infections, while there are not enough stocks of ciprofloxacin, cefotaxime, ceftriaxone and meropenem in many units. In fact, ampicillin, or its alternative amoxicillin, is still recommended by the Military Medicine, Ministry of National Defense of Vietnam in an internal descriptive document released in 2008 as an accepted therapy for treatment and prevention of bacterial meningitis in military units at the dose of 1000 mg twice daily in five consecutive days when there is no immediately available stock of other recommended antibiotics.

Complete resistance was observed against chloramphenicol, notably to an extremely high MICs of 256. To our knowledge, MIC value at 256 μg/ml against chloramphenicol observed in DuyDNT isolate is the highest to date, about 33% higher than the highest MIC recorded before [31].

### Sequencing, assembly and annotation

**Fig. 1:**
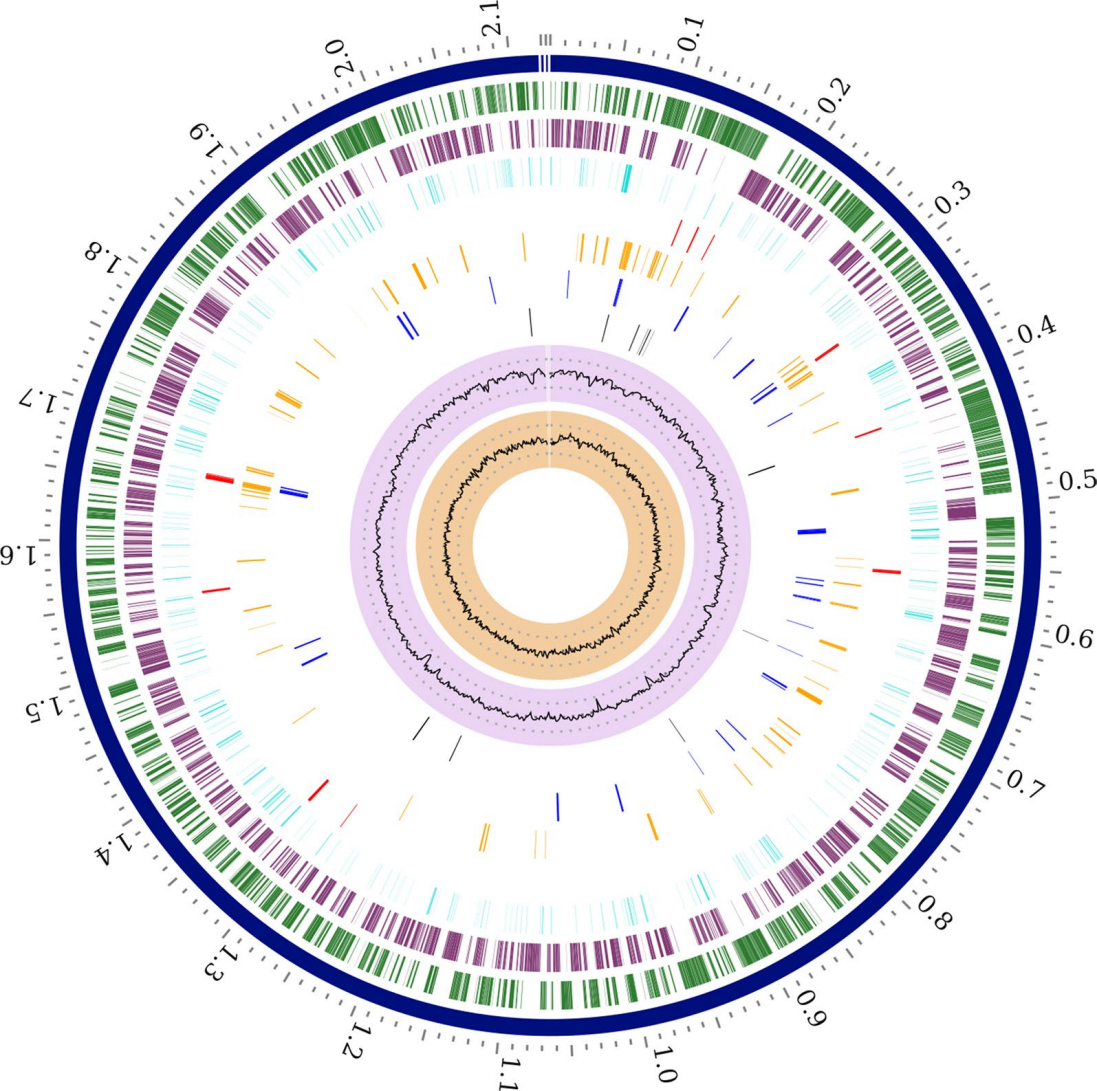
Circular view of the genome of *N. meningitidis* DuyDNT isolate generated by PATRIC showing the physical map of its significant features. From outside in: Order of contigs (shown in navy); distribution of coding sequences in plus and minus strands (shown in green and purple, respectively); distribution of noncoding elements along the chromosome (shown in blue); distribution of genes involved in antibiotic resistance (shown in red); distribution of other virulence genes (shown in orange); distribution of genes encoding transmembrane proteins (shown in dark blue); distribution of genes encoding drug targets (shown in black); distribution of GC content along plus and minus strands (most inner two circles)

From a total of 1,382,993,404 read bases, after filtering, the draft genome of *N. meningitidis* DuyDNT isolate was assembled containing 2,118,198 bps ordered into 112 contigs, which were then joined into 6 scaffolds by the MeDuSa platform using *N. meningitidis* MC58 genome sequence as reference (Fig. 1). Both genome size ( ~ 2.1 Mb) and GC content (51.17%) matched closely the average genome size and GC content of the *Neisseria* genus [32]. Genome annotation by PATRIC revealed 2451 protein coding sequences from the DuyDNT isolate that encoded for 1725 functionally assigned proteins plus 726 hypothetical ones. In total, 55 known proteins that function as virulence factor were annotated, along with 12 drug targets and 14 associated with antibiotic resistance. Besides protein coding sequences, the genome of isolate DuyDNT also encoded for 49 tRNAs and 3 rRNAs. Assembly data and genomic sequence have been deposited in NCBI Genbank under accession number RPSF00000000.

### Identification of antibiotic resistance genes

To identify genes related to antibiotic resistance from the genomic sequence of DuyDNT isolate, we submitted the sequence to CARD [33-35] as well as analyzed annotation results from PATRIC. A list of 15 coding sequences that perfectly or strictly matched antibiotic resistance-related genes in CARD database is presented in Table 3, together with reference sequence ID (source ID), predicted gene and protein products, and respective identity. Identified sequences confirmed results from antibiotic susceptibility test above, namely *penA* [36] and *catP/D* [37] that conferred resistance to ampicillin and chloramphenicol, respectively. Additionally, a mutant form of *rpoB* that loosely matched the known mutant of *rpoB* [38] was identified that might be the contributing factor to reduced susceptibility to ripamficin of DuyDNT (CARD analysis).

**Table 3:** Resistance-associated genes identified in *N. meningitidis* DuyDNT isolate

PATRIC ID	Source ID	Resistance Gene	Antibiotic class	Identity
fig|487.2031.peg.475	NP_273462.1	*penA*	Beta-lactam resistance	96
fig|487.2031.peg.1487	AAB51421.1	*catP/catD*	Chloramphenicol resistance	100
fig|487.2031.peg.154	AAA50993.1	*Tufa*	Elfamycin resistance	84
fig|487.2031.peg.138	AAA50993.1	*Tufa*	Elfamycin resistance	84
fig|487.2031.peg.620	AAV85982.1	*macB*	Erythromycin resistance	98
fig|487.2031.peg.619	AAV85981.1	*macA*	Erythromycin resistance	96
fig|487.2031.peg.1534	YP_207769.1	*gyrA*	Fluoroquinolone resistance	97
fig|487.2031.peg.1792	YP_208330.1	*ngo1259*	Fluoroquinolone resistance	97
fig|487.2031.peg.186	WP_002215466.1	*oxyR*	Isoniazid resistance	100
fig|487.2031.peg.1920	NP_274719.1	*farA*	Multidrug efflux	98
fig|487.2031.peg.359	NP_273368.1	*mtrR*	Multidrug efflux	98
fig|487.2031.peg.1919	NP_274718.1	*mtrC*	Multidrug efflux	99
fig|487.2031.peg.358	NP_273367.1	*farB*	Multidrug efflux	99
fig|487.2031.peg.1921	YP_002002225.1	*mtrD*	Multidrug efflux	97
fig|487.2031.peg.155	YP_208874.1	*rpsJ*	Tetracycline resistance	100

Besides these, variants with 84–100% identity to known mutations conferring resistance against other antibiotics such as elfamycin, erythromycin, fluoroquinolone, isoniazid, tetracycline or multi-drug target were identified from DuyDNT genome. All identified resistance genes have been previously reported in *N. meningitidis* and *N. gonorrhoeae*, however the variants of *ngo1259* and *mtrD* found in DuyDNT genome were observed for the first time. The presence of such wide repertoire of sequences involved in antibiotic resistance in DuyDNT genome is likely the result of both the high transformable nature of meningococcal B and excessive use of antibiotics in Vietnam. Sequence of the identified variants might contribute important information to deduce antibiotic resistance mechanisms in *N. meningitidis.*

### Epidemiological characterization of DuyDNT isolate

Additional antigenic typing of DuyDNT isolate were done using sequences extracted from WGS data. Antigenic determinants’ fine structure of was analyzed using PubMLST finetyping antigens scheme that included two variable regions of gene *proA* (VR1 and VR2) and another one of gene *FetA* (VR)*.* The resulting profile for DuyDNT isolate was VR1: 22–25, VR2: 14–32, VR: F4-6 (Table 1), which was a novel sequence type that varied from other isolates in the database by at least two out of three tested alleles.

We then performed Bexsero Antigen Sequence Typing (BAST) to estimate the likelihood of DuyDNT isolate to be covered by the two recently developed vaccines against *N. meningitidis* B, Bexsero^®^ and Trumenba^®^ [39, 40]. Notably, all allelic variants found in DuyDNT’s BAST profile (fHBP: 31, NHBA: 16, NadA: 0, PorA VR1: 22–25, PorA VR2: 14–32) were predicted to have no reactivity with either Bexsero^®^ or Trumenba^®^, suggesting the potential for no protectiveness of these vaccines against this isolate. Taken together, epidemiological characteristics inferred from genomic sequence of DuyDNT isolate showed significant distinctiveness from other global *N. meningitidis* strains. These results highlight the need for an update epidemiological surveillance of IMD to effectively support vaccination strategy in Vietnam.

### Features of DuyDNT isolate’s genome

Repetitive DNA sequences are important features of *N. meningitidis* genomes. They result in genome modification and are involved in gene expression regulation, thus playing important roles in *N. meningitidis* virulence and host immune invasion [41, 42]. The top three most abundant repetitive DNA motifs of the *Neisseria* genus known to date are DNA uptake sequence (DUS), DSR3 elements and Correia (CE) elements [43]. DUS is essential for DNA transformation, while DSR3 and CE are often found at phage integration sites and promoter sequences, respectively [41, 43–46].

A search for repetitive DNA sequences in the genome of DuyDNT isolate by fuzznuc (EMBOSS) revealed 1864 copies of DUS, 828 DSR3 elements and 198 Correia elements among 151 repetitive motifs found (Table 4). The composition of major repetitive motifs in DuyDNT genome is similar to that of MC58, a reference strain for *N. Meningitidis* B, with the only notable exception of DSR3. DSR3 was over-represented in the genome of MC58 isolate by ~ 67% compared to that of DuyDNT genome. Among species of *Neisseria*, DUS appeared to be similarly presented, while both DSR3 and CE were two to three fold over-represented in *N. meningitidis* compared to *N. gonorrhoeae* and *N. lactamica*. A linear correlation between the number of DUS and DNA uptake efficiency was reported in *N. gonorrhoeae*, thus fewer DSR3 copies might indicate a lesser extent for the Vietnamese isolates’ genomes to incorporate external DNA sequences, though the same observation was not made in *N. Meningitidis* [47]. On the other hand, the Vietnamese isolate appeared to carry more AG-mucDUS copies compared to that of MC58, thus might allowing it to exchange DNA more frequently with species of *Neisseria* that predominantly possess this motif such as *N. mucosa, N. polysaccherea* or *N. cinerea* [43].

**Table 4:** Frequency of prominent repetitive DNA sequences in DuyDNT genome

Motifs	Repetitive sequences	*N. meningitidis* DuyDNT	*N. meningitidis* MC58	*N. lactamica* Y92–1009	*N. gonorrhoeae* NCCP11945
DUS	GCCGTCTGAA	1864	1935	2247	445
AT-DUS (eDUS)	ATGCCGTCTGAA	1449	1477	1718	1520
vDUS	GTCGTCTGAA	173	165	90	120
AG-DUS	AGGCCGTCTGAA	181	214	262	192
AG-mucDUS	AGGTCGTCTGAA	102	88	45	83
veDUS	ATGTCGTCTGAA	20	19	8	9
DSR3	ATTCCCNNNNNNNNGGGAAT	828	1378	454	430
Correia	ATAG[CT]GGATTAACAAAAATCAGGAC	166	181	50	78
	TATAG[CT]GGATTAAATTTAAACCGGTAC	1	0	1	23
	TATAG[CT]GGATTAACAAAAACCGGTAC	5	8	17	40
	TATAG[CT]GGATTAAATTTAAATCAGGAC	26	24	17	21
Total^a^		151	209	152	131

^a^Performed by tandem repeat finders [29]

The presence of prophages is another important feature of a genome sequence, since it indicates regions for potential horizontal DNA transfer. Using PHASTER, we looked for prophage regions in the genome of DuyDNT isolate, as well as MC58 (Table 5). Both genomes contained two prophage regions of different intactness originated from three known phages. One region, originated from PHAGE_Haemop_SuMu_NC_019455, was shared by both tested genomes; though only MC58 carried the intact region, while DuyDNT isolate retained ~ 37% of the region. The second prophage region, an intact prophage originated from PHAGE_Pseudo_YMC11/02/R656_NC_028657, presented in DuyDNT isolate while completely absent in the genome of MC58. In contrast, only MC58 carried a partial genome of PHAGE_Burkho_BcepIL02_NC_012743, about 24.6 kb that encoded for 29 proteins.

**Table 5:** Prophage regions in genomes of DuyDNT and MC58

Isolate	Region 1				Region 2				
	Region length (kb)	Total proteins	Status	Closest known phage	Region length (kb)		Total proteins	Status	Closest known phage
DuyDNT	35.3	41	Intact	PHAGE_Pseudo_YMC11/02/R656_NC_028657	12		12	Partial	PHAGE_Haemop_SuMu_NC_019455
MC58	24.6	29	Partial	PHAGE_Burkho_BcepIL02_NC_012743	32.8		49	Intact	

A Blast search revealed that two prophages PHAGE_Pseudo_YMC11/02/R656_NC_028657 and PHAGE_Burkho_BcepIL02_NC_012743 were commonly found in genomic sequences of meningococcus but no other species of *Neisseria*. Contrary, PHAGE_Haemop_SuMu_NC_019455 was found most prominently in *N. meningitidis*, but also in *N. gonorrhoeae, N. polysaccharea* and *N. lactamica.* Taken together, both repetitive DNA and prophage features highlighted the genome flexibility shared among *N. meningitidis* serotype B, while also emphasizing potential for genome modifications and expression modification observed only in the tested Vietnamese isolate.

## Conclusions

In this study, we have described and analyzed for the first time the genome of a drug-resistant invasive *N. meningitidis* B isolate from a military unit in Vietnam. This isolate, designated DuyDNT, showed the highest MIC (256 μg/ml) against chloramphenicol known to date, while also displaying diminished susceptibility toward ampicillin and rifampicin, the latter is still widely used for prophylaxis in numerous clinical units in Vietnam. Multi-locus sequence typing for the isolate revealed a rare sequence type (ST 13074) found only in two other isolates from Vietnam identified by our laboratory. DuyDNT genome was sequenced and assembled into 6 scaffolds of 2,118,198 bps, yielding a total of 2451 protein coding sequences, as well as 49 tRNAs and 3 rRNAs. A search for genes involved in antibiotic resistances in the genome of DuyDNT recovered 15 coding sequences that might contribute to antibiotic resistance and reduced susceptibility. Among these some novel variants were found that potentially render the isolate even broader drug resistance. Additional epidemiologic characterization revealed DuyDNT has a unique antigenic finetype (VR1: 22–25, VR2: 14–32, VR: F4–6) and Bexero^®^ BAST type (fHBP: 31, NHBA: 16, NadA: 0, PorA VR1: 22–25, PorA VR2: 14–32), the latter predicted this isolate was not covered by either Bexsero^®^ or Trumenba^®^, the two most recently developed vaccines against meningococcal B. Genomic composition of DuyDNT isolate consisted of various repetitive DNA sequences and prophage regions with some features unique for just the Vietnamese isolate, pointing toward a flexible genome capable of exchanging DNA with other species of *Neisseria*. Altogether, our results illustrated the usefulness of WGS analysis for epidemiological and antibiotic resistance surveillance of IMD. Our study also highlights the need for a more comprehensive study of the diversity among *N. meningitidis* isolates in Vietnam and a standard molecular characterization scheme in order to accurately monitor antibiotic resistance of *Neisseria* species among military units as well as support an effective IMD vaccination strategy in Vietnam.
